# Application of Artificial Intelligence and Machine Learning in Diagnosing Scaphoid Fractures: A Systematic Review

**DOI:** 10.7759/cureus.47732

**Published:** 2023-10-26

**Authors:** Chijioke Orji, Maiss Reghefaoui, Michell Susan Saavedra Palacios, Priyanka Thota, Tariladei S Peresuodei, Abhishek Gill, Pousette Hamid

**Affiliations:** 1 Trauma and Orthopaedics, California Institute of Behavioral Neurosciences & Psychology, California, USA; 2 Internal Medicine, University of Debrecen, Debrecen, HUN; 3 College of Medicine, University of Cuenca, Cuenca, ECU; 4 Internal Medicine, California Institute of Behavioral Neurosciences & Psychology, California, USA; 5 Medicine, Kent and Canterbury Hospital, Canterbury, GBR; 6 Neurology, California Institute of Behavioral Neurosciences & Psychology, California, USA

**Keywords:** machine learning, radiology, orthopaedics, scaphoid fracture, artificial intelligence

## Abstract

The integration of artificial intelligence (AI) in healthcare has sparked interest in its potential to revolutionize medical diagnostics. This systematic review explores the application of AI and machine learning (ML) techniques in diagnosing scaphoid fractures, which account for a significant percentage of carpal bone fractures and have important implications for wrist function. Scaphoid fractures, common in young and active individuals, require an early and accurate diagnosis for effective treatment. AI has the potential to automate and improve the accuracy of scaphoid fracture detection on radiography, aiding in early diagnosis and reducing unnecessary clinical examinations. This systematic review discusses the methods used to identify relevant studies, including search criteria and quality assessment tools, and presents the results of the selected studies. The findings indicate that AI-driven methods can improve diagnostic accuracy, reducing the risk of missed fractures and complications. AI assistance can also alleviate the workload of medical professionals, improving diagnostic efficiency and reducing observer fatigue. However, challenges such as algorithm limitations and the need for continuous refinement must be addressed to ensure reliable fracture identification. This review underscores the clinical significance of AI-assisted diagnostics, especially in cases where fractures may be subtle or occult. It emphasizes the importance of integrating AI into medical education and training and calls for robust data collection and collaboration between AI developers and medical practitioners. Future research should focus on larger datasets, algorithm improvement, cost-effectiveness assessment, and international partnerships to fully harness the potential of AI in diagnosing scaphoid fractures.

## Introduction and background

Will artificial intelligence (AI) replace doctors? Is healthcare heading in the direction where a radiologist, an orthopedic surgeon, or an emergency department physician will not be needed to interpret the radiographic images of a patient with a suspected scaphoid fracture?

AI provides tremendous potential in healthcare, not only by automating some of the problem-solving tasks performed by doctors and other medical professionals but also by making faster and better judgments and using problem-solving strategies that humans alone cannot. In recent years, there has been much interest in using AI to diagnose scaphoid fractures. Several studies have been conducted to investigate the potential of AI, notably convolutional neural networks (CNNs), in increasing the accuracy and efficiency of identifying scaphoid fractures on radiography [1-6]. A CNN is a branch of the machine learning (ML) subclass, a branch of AI. CNNs are utilized in image recognition and processing as they are specially built to process pixel data [7].

Scaphoid fractures comprise 82%-89% of all carpal bone fractures and 2%-7% of all fractures with catastrophic repercussions owing to their essential role in wrist function. It is most common in young and active males [8]. Therefore, adequate diagnosis at the point of care is of utmost importance. Early detection of suspected scaphoid fractures allows for faster treatment when necessary. In contrast, the quick elimination of suspected fractures minimizes undue immobility and needless additional clinical examinations for individuals who are not injured. The ideal diagnosis algorithm would ensure no fractures are overlooked; despite substantial research into secondary imaging modalities, a straightforward and cost-effective diagnostic methodology is yet to be created [1,9-12].

This systematic review will give us exposure to the different studies in which AI has been applied to diagnose scaphoid fractures. We will assess the possibility of using AI in clinical situations and its applicability.

## Review

Methods

This systematic review used the Preferred Reporting Items for Systematic Reviews and Meta-analyses (PRISMA) guideline [13].

Database

A systematic search was conducted on PubMed, Embase, Google Scholar, and OrthoSearch from the beginning of the respective databases till the 31st of May, 2023.

Search Strategy

The following search keywords were used on the databases: "Machine learning" [tw] OR "Artificial intelligence" [tw] OR "deep learning" [tw] OR "deep convolutional neural network" [tw] OR "large language model" [tw] OR "deep neural network" [tw] AND orthopedic*[tw] OR orthopaedic*[tw] OR "musculoskeletal disorder*" [tw] OR bone [tw] OR fracture [tw] OR "joint replacement" [tw] OR arthroplasty [tw] AND diagnos*[tw] OR detection [tw] OR prognosis [tw] OR forecast [tw] OR classification [tw] OR accuracy [tw] OR evaluat*[tw] OR identify [tw].

Screening

Two reviewers independently screened the articles' titles and abstracts for eligibility after removing duplicates. Where there was no consensus, a third reviewer, who was not involved in the initial review, decided the eligibility.

Inclusion Criteria

We included research using AI or ML approaches for diagnosing scaphoid fractures. Human participants and genuine clinical data must be used in the studies, published in peer-reviewed publications, and available in English. We evaluated studies that offer detailed explanations of the AI/ML methodologies used to guarantee clarity and relevance. In addition, studies that disclose essential outcomes such as sensitivity, specificity, and area under the curve were included (AUC).

Exclusion Criteria

We excluded papers that did not use AI or ML approaches to diagnose scaphoid fractures, including research that used animal models or in vitro techniques. Review articles, opinion pieces, editorials, and conference abstracts were also not eligible. Studies that did not provide details of the AI/ML methodologies used were disqualified. Non-English language articles were excluded from consideration. Additionally, papers focusing on non-AI/ML approaches for diagnosing scaphoid fractures were omitted from our analysis. Finally, papers that did not provide appropriate outcome measures or statistical data were excluded from consideration.

Quality Assessment Tools

Two reviewers assessed each selected article independently for quality using the modified Quality Assessment of Diagnostic Accuracy Studies (QUADAS-2) [14]. Data was extracted and stored using standardized forms on an electronic database (Microsoft Excel v.16.74; Microsoft Inc., Redmond, WA). The two reviewers held a consensus meeting to settle the discordance in quality assessment and data extraction.

Results

A total of 3884 articles were found using a keyword search through four databases; 234 duplicates were removed, and 3628 articles were excluded following title and abstract screening. We retrieved full-text articles from all the remaining 22 papers, and these underwent a full-text review using the inclusion/exclusion criteria, with 14 articles eliminated for multiple reasons. All remaining articles underwent quality assessment using the QUADAS-2 statement and findings outlined in Table 1.

**Table 1:** Quality assessment for risk of bias using the QUADAS-2 statement L: Low risk of bias; U: Unclear risk of bias; H: High risk of bias; QUADAS-2: Quality Assessment of Diagnostic Accuracy Studies.

Study	Risk of Bias				Applicability Concerns		
	Patient Selection	Index Test	Reference Standard	Flow and Timing	Patient Selection	Index Test	Reference Standard
Langerhuizen et al., 2020 [15]	L	L	L	L	U	L	L
Yoon et al., 2021 [5]	L	L	L	U	L	L	L
Hendrix et al., 2021 [3]	L	L	L	L	L	L	L
Ozkaya et al., 2022 [16]	L	U	L	L	L	L	L
Yoon et al., 2022 [17]	H	L	L	L	L	L	H
Yang et al., 2022 [18]	L	L	H	L	L	L	L
Hendrix et al., 2023 [19]	L	L	L	L	L	U	L
Singh et al., 2023 [20]	L	L	L	L	L	L	L

**Figure 1:**
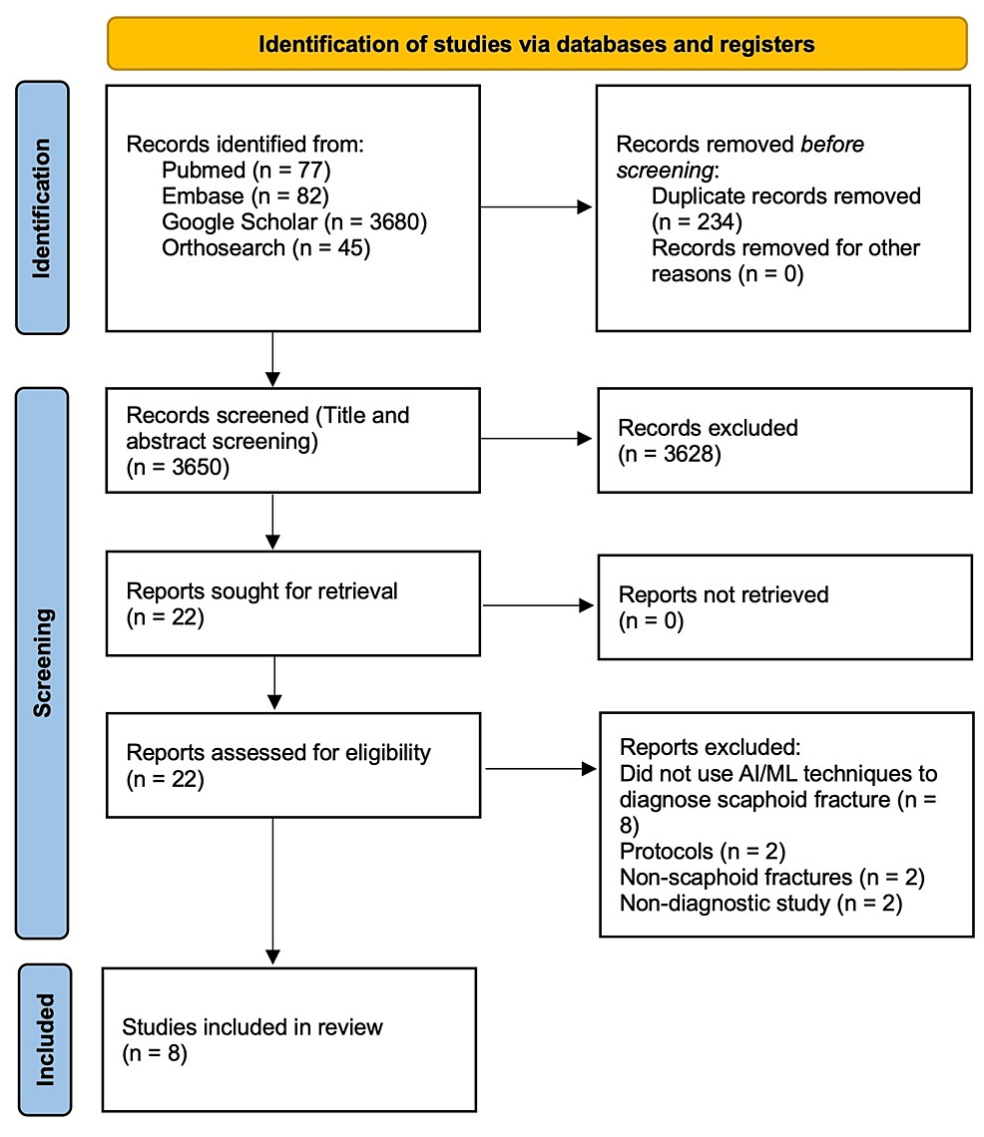
PRISMA flow diagram PRISMA: Preferred Reporting Items for Systematic Reviews and Meta-analyses.

All eight articles were included in the systematic review. The PRISMA flow diagram is seen in Figure 1.

Outcome Measure

Seven studies [3,5,15-17,19,20] used either computed tomography (CT) or magnetic resonance imaging (MRI) scans or both to establish the ground truth of a scaphoid fracture. In contrast, one study [18] did not state the source of their ground truth. Five articles described detecting apparent and occult fractures using convoluted neural networks (CNN) [5,15,17,18,20], and the remaining three only classified scaphoid fractures as apparent or no fracture [3,16,19]. Seven of the included studies reported sensitivity, specificity, and area under the receiver operating curve (AUC) of the models used at ranges of 72 - 92%, 60 - 93%, and 0.77 - 0.95, respectively. Details of extracted data are shown in Table 2.

**Table 2:** Study characteristics CNN: Convoluted neural network; CT: Computed tomography; MRI: Magnetic resonance imaging; AUC: Area under the receiver operating curve; ED: Emergency department; DCNN: Deep convolutional neural network.

Authors	Title	Number of Patients	Number of Images	Algorithm/Model	Reference Standard	Sensitivity	Specificity	AUC	Summary
Langerhuizen et al., 2020 [15]	Is deep learning on par with human observers for detection of radiographically visible and occult fractures of the scaphoid?	150	300	CNN	CT/MRI	84%	60%	0.77	Including patient demographic information does not affect diagnostic performance. Orthopedic surgeons had higher specificity than the algorithm, but accuracy and sensitivity were not substantially different.
Yoon et al., 2021 [5]	Development and validation of a deep learning model using convolutional neural networks to identify scaphoid fractures in radiographs	775	1039	CNN (EfficientNetB3)	CT/MRI	78%	84%	0.87	The developed CNN achieved radiologist-level performance detecting scaphoid bone fractures on conventional hand, wrist, and scaphoid radiographs.
Hendrix et al., 2021 [3]	Development and validation of a convolutional neural network for automated detection of scaphoid fractures on conventional radiographs	7729	11838	CNN	CT	First stage – 87.1%. Second stage – 79.0%.	First stage –92.1%. Second stage – 71.6%.	First stage –0.955. Second stage – 0.810.	These findings suggest that DCNNs can be trained to detect fractures of small bones, such as scaphoids, reliably, and may assist with radiographic detection of occult fractures that are not visible to human observers.
Ozkaya et al., 2022 [16]	Evaluation of an artificial intelligence system for diagnosing scaphoid fracture on direct radiography	390	390	CNN	CT	76%	92%	0.840	Experienced orthopedic specialists had the best diagnostic performance according to AUC; the CNN had similar performance with less experienced orthopedic specialists, but it was better than the ED physician.
Yoon et al., 2022 [17]	Can a deep learning algorithm improve detection of occult scaphoid fractures in plain radiographs? A clinical validation study	–	15	CNN	CT/MRI	87%	76%	–	CNN assistance improves physician diagnostic sensitivity and specificity and interobserver agreement for the diagnosis of occult scaphoid fractures.
Yang et al., 2022 [18]	Scaphoid fracture detection by using convolutional neural network	–	360	CNN (ResNet 152)	Not stated	78.9%	90%	0.920	The CNN effectively detects scaphoid fractures and has potential for future applications.
Hendrix et al., 2023 [19]	Musculoskeletal radiologist-level performance by using deep learning for detection of scaphoid fractures on conventional multiview radiographs of hand and wrist	4796	19111	CNN (DenseNet-121)	CT	72%	93%	0.88	The CNN diagnoses scaphoid fractures on traditional multiview X-rays at the level of five competent musculoskeletal radiologists, potentially cutting their reading time in half.
Singh et al., 2023 [20]	Automated detection of scaphoid fractures using deep neural networks in radiographs	–	525	CNN	MRI	Two class classification – 92%. Three class classification – 85%.	Two class classification – 88%. Three class classification – 91%.	Two class classification – 0.95. Three class classification – 0.88.	Model excelled in occult scaphoid fracture detection.

Discussion

Integrating AI and ML techniques in medical diagnostics has shown remarkable potential in revolutionizing healthcare practices. This systematic review of the application of AI and ML in diagnosing scaphoid fractures reveals a growing body of research to enhance accuracy, efficiency, and clinical decision-making in this domain.

The risk of fracture non-union rises when concealed scaphoid fractures are not detected. Untreated non-unions can lead to intercarpal and radiocarpal degeneration, osteonecrosis, carpal instability, and scaphoid non-union advance collapse (SNAC) [21]. Advanced imaging, specifically MRI, is essential to diagnose a suspected scaphoid fracture early. MRI is the gold standard imaging modality for detecting acute scaphoid fractures, with 94.2% sensitivity and 97.7% specificity [22]. Therefore, developing an AI with similar sensitivity and specificity will increase the efficiency and accuracy of diagnosing scaphoid fractures, possibly decrease the cost associated with using advanced imaging, and may be more accessible in remote areas with less specialist service or access to advanced imaging.

This systematic review demonstrates several advantages of using AI/ML methods for scaphoid fracture diagnosis. One of the primary advantages is the potential to improve diagnostic accuracy. Traditional methods may occasionally lead to misdiagnosis or missed fractures due to complex anatomical structures and subtle fractures in the scaphoid bone [21,22]. AI algorithms, such as CNNs, have demonstrated their ability to accurately identify visible and occult fractures [5,17,20]. The studies consistently report high sensitivity and specificity levels, suggesting that AI-based systems can be reliable diagnostic tools in clinical settings.

Furthermore, AI-driven diagnosis has the potential to significantly reduce the workload of radiologists and other medical professionals involved in fracture detection. AI assistance has been shown to enhance interobserver agreement among radiologists and save reading time [19]. Also, nearly 40% of all inpatient imaging studies require prompt attention [23]. Such high-volume, urgent radiological reports can lead to observer fatigue, reducing diagnostic ability, especially in cases like scaphoid fractures, where radiographic identification is already tricky. Pre-processing radiographs with DCNNs might reduce observer fatigue and missed fractures [5], which could lead to faster diagnosis and treatment decisions, ultimately improving patient outcomes.

Despite the promising findings, challenges remain in implementing AI-assisted diagnosis in clinical settings. While the CNNs demonstrated good performance, they still faced limitations. For instance, some studies noted that the algorithms had difficulty detecting fractures apparent to human observers [3,14,17], highlighting the need to continuously refine these algorithms to ensure they can accurately identify fractures across various presentations. This also illustrates that currently, CNNs and AI-supported diagnostics serve as complementary aids to radiologist assessments, offering the potential to enhance the efficiency of diagnoses, while recognizing the ongoing necessity for concurrent human evaluation.

There is considerable evidence to suggest that AI aid might increase musculoskeletal radiologists' diagnostic efficiency [19]. AI techniques such as CNNs can improve physician diagnostic performance when identifying occult scaphoid fractures on plain radiographs. The interobserver agreement increased when CNN was used to identify occult scaphoid fractures on radiographs, highlighting the importance of integrating AI as a supportive tool rather than replacing clinical expertise [17,20]. In recognizing common fractures such as the neck of the femur, proximal humerus, distal radius, and ankle fractures, AI models were demonstrated to be as accurate as human observers. Furthermore, AI models outperformed humans in identifying proximal humerus and hip fractures [24]. Collaboration between AI and medical professionals can lead to synergistic outcomes, combining the strengths of both AI and human judgment.

The findings of this systematic review have significant clinical implications. Missed diagnosis is a severe problem that has affected emergency departments for some years, with about three out of every four claims showing that the radiologist misinterpreted the case [25,26]. AI-powered diagnostic tools can potentially improve the accuracy of scaphoid fracture detection, especially in cases where fractures might be subtle or occult [6,17,18,20]. Rapid and accurate diagnosis is crucial for effective treatment planning and patient care, particularly given the functional importance of the scaphoid bone [8,10,22].

As technology advances and AI algorithms continue to evolve, it is essential to consider their integration into medical education and training. Healthcare professionals should be equipped with the skills to utilize AI tools effectively, interpret their results, and make informed decisions based on AI-generated outputs [27,28]. Moreover, this systematic review emphasizes the importance of robust data collection and sharing to enhance the development and validation of AI models in medical diagnostics.

This systematic review provides helpful information; however, significant limitations should be noted. The evaluation is limited in time and may not include the most recent breakthroughs in AI technology. Furthermore, the review focuses on studies published in English, which may exclude significant research undertaken in other languages.

More research is needed to overcome the shortcomings identified in recent studies. Larger datasets should be used to improve the algorithm's detection of less prevalent fracture patterns. Collaboration between AI developers and medical practitioners is critical for developing these algorithms and ensuring that clinical demands and standards are followed [29,30]. Furthermore, the cost-effectiveness of creating and deploying AI systems should be extensively assessed.

## Conclusions

In conclusion, using AI and ML, particularly CNNs, in diagnosing scaphoid fractures shows the potential for enhancing diagnostic accuracy and efficiency. While challenges remain, the evidence suggests that AI-driven diagnostic tools can enhance accuracy, efficiency, and collaboration among medical professionals. However, issues such as the need for additional algorithm improvement remain. Collaborations between AI developers and medical experts will be critical in reaching the full potential of AI-assisted diagnostics for scaphoid fractures.
